# The Effect of Synergistic Approaches of Features and Ensemble Learning Algorith on Aboveground Biomass Estimation of Natural Secondary Forests Based on ALS and Landsat 8

**DOI:** 10.3390/s21175974

**Published:** 2021-09-06

**Authors:** Chunyu Du, Wenyi Fan, Ye Ma, Hung-Il Jin, Zhen Zhen

**Affiliations:** 1 School of Forestry, Northeast Forestry University, Harbin 150040, China; duchunyu402@163.com (C.D.); fanwy@163.com (W.F.); maye799535410@163.com (Y.M.); jhicoco@nefu.edu.cn (H.-I.J.); 2 Jilin Forestry Research Institute, Jilin 132013, China; 3 Key Laboratory of Sustainable Forest Ecosystem Management-Ministry of Education, Northeast Forestry University, Harbin 150040, China; 4 Faculty of Forest Science, Kim Il Sung University, Pyongyang 999093, Democratic People’s Republic of Korea

**Keywords:** ensemble learning, machine learning, feature extraction, AGB, NSFs

## Abstract

Although the combination of Airborne Laser Scanning (ALS) data and optical imagery and machine learning algorithms were proved to improve the estimation of aboveground biomass (AGB), the synergistic approaches of different data and ensemble learning algorithms have not been fully investigated, especially for natural secondary forests (NSFs) with complex structures. This study aimed to explore the effects of the two factors on AGB estimation of NSFs based on ALS data and Landsat 8 imagery. The synergistic method of extracting novel features (i.e., *COLI*1 and *COLI*2) using optimal Landsat 8 features and the best-performing ALS feature (i.e., elevation mean) yielded higher accuracy of AGB estimation than either optical-only or ALS-only features. However, both of them failed to improve the accuracy compared to the simple combination of the untransformed features that generated them. The convolutional neural networks (CNN) model was much superior to other classic machine learning algorithms no matter of features. The stacked generalization (SG) algorithms, a kind of ensemble learning algorithms, greatly improved the accuracies compared to the corresponding base model, and the SG with the CNN meta-model performed best. This study provides technical support for a wall-to-wall AGB mapping of NSFs of northeastern China using efficient features and algorithms.

## 1. Introduction

The Asian temperate mixed forest in northeastern China is one of the three major temperate mixed forests in the world (i.e., northeastern North America, Europe, and East Asia) [1], which is of great strategic importance to the carbon trading of China. The forests of Northeast China have experienced three periods of excessive timber harvesting in the last century, including the period of Russian and Japanese aggression (1896–1945), the period of encouraging excessive harvesting for timber production (1950–1977), and the period of national economic reforms and the broadening of international relations (1978–1998) [2]. The excessive logging and neglected cultivation of forests nearly exhausted exploitable forest reserves in the region [3]. Since the Natural Forest Conservation Program (NFCP) was put into practice in 1998, there was a profound shift in focus from timber production to environmental protection by rehabilitating damaged forest ecosystems, afforesting desertified and degraded areas, and banning logging in natural forests [2]. In this context, natural secondary forests (NSFs) are gradually expanding and gaining importance. NSFs, which account for as much as 70% of the forests of northeastern China, refer to as the natural-regeneration forests after stand-replacing disturbances of primary forests by anthropogenic activities or by extreme natural events [4,5]. Nowadays, the NSFs of northeastern China are gradually recovering from the excessive logging of the 20th century, which led to an extraordinary reduction in the quality of the forest ecosystem. NSFs are of significance to China not only for timber supply, but also for a vital reservoir of biodiversity, potential carbon sequestration, a destination of ecological tourism, and a broad ecological shelter for northeastern China [2].

The accurate estimation of forest aboveground biomass (AGB) has a critical effect on the understanding of forest quality and recovery in the NSFs of northeastern China. AGB is defined as the dry mass of live or dead matter from tree or shrub life forms, typically expressed as a mass per area density (e.g., Mg/ha) [6]. In general, AGB could be obtained by (1) direct harvest method; (2) allometric equation-based method; (3) biomass expansion factor (BEF)-based method; (4) process-based biogeochemical modeling; (5) remote sensing-based estimation method. Although the direct harvest method is the most exact among these methods, it is time-consuming, destructive, and labor-intensive. It is only suitable for AGB estimation of a small area or of individual trees with a small sample size [7] and is usually applied as reference data to establish allometric equations of AGB (e.g., [8,9]). An allometric equation-based method is more flexible and feasible than the direct harvest method to estimate AGB on both individual tree and plot levels. A variety of allometric equations are developed for diverse tree species by modeling the relationship between AGB and various physical parameters of trees, such as diameter at breast height (DBH), tree height, crown diameter, etc. (e.g., [8,10,11,12,13]). Similar to an allometric equation-based method, the BEF-based method applied BEF defining as the ratio of all stand biomass to growing stock volume to convert timber volume to biomass [14]. However, the allometric equation-based and BEF-based methods are still time-consuming and expensive because both of them are based on the acquisition of field measurements (such as DBH, tree height), and still limited to plot-level or individual tree-level AGB estimations. Process-based biogeochemical models consider the processes including photosynthesis, absorption, and carbon allocation, and generally couple biology, soil, climate, hydrology, and anthropogenic effects [15]. To some degree, these models could improve the conventional, point-based estimation of biomass over large areas [16]. However, the high uncertainties in biomass estimation due to constraints in data source, spatial resolution, homogeneous assumption, and inaccuracy of models greatly limit the usage of process-based biogeochemical models [15]. The remote sensing-based method is exceedingly appealing for estimating forest biomass on a large scale (e.g., local, regional or global) because of its unique characteristics such as repetitive data acquisition, large coverage, digital format, and so on [15], and of the capability of providing spatially explicit AGB estimates for every pixel location, instead of only the mean or total biomass within a given inventory unit [17,18]. Nowadays, it becomes the most commonly used method for large-scale AGB estimation [19,20,21].

In the last three decades, researchers have attempted a variety of remotely sensed data sources to estimate AGB. With a relatively long history of data availability, optical satellite imagery (such as Landsat, MODIS, etc.) has become a primary data source for biomass estimation (e.g., [22,23,24,25]). In particular, Landsat series satellite imagery is the most commonly used data source for AGB estimation (e.g., [26,27,28,29,30]), mainly because of the continuous, long-term, medium spatial resolution, and cross-calibrated data for global surface observations, and free access policy [31]. However, it is of significance to notice the data saturation in Landsat imagery, which refers to the phenomenon that spectral reflectance values are not sensitive to the change in biomass of mature forest or advanced successional forests even if AGB varies significantly [32,33]. For example, Steininger [34] found that the canopy reflectance in Landsat imagery saturates when the AGB approaches 15 kg/m^2^ or over 15 years of age in Brazilian tropical secondary forests. Zhao et al. [33] examined the saturation values in Landsat imagery for different vegetation types in a subtropical region, and found the AGB saturation values for pine forest, mixed forest, Chinese fir forest, broadleaf forest, bamboo forest, and shrub were 159, 152, 143, 123, 75, and 55 Mg/ha, respectively. Data saturation in optical imagery like Landsat significantly lowers the accuracy and increases the uncertainties of AGB estimation [15]. Data saturation still exists in RADAR (Radio Detection and Ranging) data like SAR (Synthetic Aperture Radar) [35]. Generally speaking, saturation values could be higher obtained by longer wavelengths (such as L and P bands) and lower by shorter wavelengths (such as C bands), and also vary for different forest structures [36]. Until now, the data saturation problem caused by remote sensing signals is still one of the biggest obstacles to applying optical imagery and RADAR data for AGB estimation [15,37,38].

Since the 1990s, it has been found that LiDAR (Light Detection and Ranging) is more advantageous than optical imagery for AGB estimation because it is more relative to tree height and produces less estimation error [39]. Meanwhile, LiDAR is unaffected by the data saturation problem, even for high AGB values (>1000 Mg/ha) [40]. Thus, LiDAR data is widely used in AGB estimation in the last two decades. According to the format of return signals, LiDAR can be classified into discrete and continuous LiDAR; according to platforms, LiDAR can be classified into spaceborne, airborne, UAV(Unmanned Aerial Vehicle), terrestrial, backpack/handheld LiDAR; according to the size of the footprint, LiDAR can be classified into small footprint (footprint size <1 m), mid footprint (footprint size: 10–30 m), and large footprint LiDAR (footprint >50 m) [41]. In recent years, Airborne Laser Scanning (ALS) data, a kind of discrete, multiple returns, and small footprint LiDAR data captured from an aerial platform, has received much scientific and operational attention for AGB estimation than any of the other remote sensing data [42]. ALS emits laser pulses towards the ground and receives the pulses reflected from the tree canopy, branches, leaves, trunk, shrub, and then ground to form a three-dimensional profile of forest structure. ALS is far more capable than optical and RADAR sensors in estimating forest parameters and is considered the premier tool for large-scale AGB estimation (e.g., [43,44,45,46,47]). It is beneficial to estimate AGB by capturing both two-dimensional spectral information of the upper canopy and three-dimensional structural information of the canopy. However, the spectral characteristics of vegetation provided by ALS are very limited since most LiDAR systems only work at a single wavelength [48]. Thus, the integration of optical imagery and ALS data has become the most promising approach for large-scale AGB estimation (e.g., [48,49,50,51,52,53]).

Features are the most direct representation or manifestation of data sources. Feature extraction and selection could greatly influence the accuracy of AGB estimation [54]. A variety of spectral-related features including band combinations, textures, diverse vegetation indices, leaf area index, fraction of vegetation cover, and so on were derived from optical imagery for AGB estimation (e.g., [29,55,56,57,58]). Similar, diverse point-based features including height statistics (e.g., mean, maximum, variance, skewness, etc.), canopy-based quantile estimators, canopy relief ratio, laser penetration rates, canopy closure, and so on were extracted from LiDAR data for AGB estimation (e.g., [25,46,52,59,60]). Some researchers directly combined optical imagery and LiDAR features (e.g., [21,61,62]) while a few of them designed novel features derived from optical imagery and LiDAR data to improve AGB estimation. For example, Zhang et al. [48] developed two novel groups of features (i.e., *COLI*1 and *COLI*2) using seven vegetation indices derived from Landsat 8 and the best-performing LiDAR variable (i.e., mean of height). The *COLI*1 and *COLI*2 were generated by the multiplication and ratio combinations of the best-performing LiDAR variable and each vegetation index, respectively. They found that the stacked sparse autoencoder network model with the combination of all *COLI*1, optical, and LiDAR features yielded the highest accuracy of AGB estimation for the coniferous and broadleaf mixed forest of southeast China. However, whether it is more efficient to use novel features extracted from both data than directly combine all features is still needed to be further investigated.

In addition to data sources and features, it is vital to establish a reliable and suitable model to estimate AGB. Currently, most remote sensing-based AGB estimation methods use data-driven empirical models, which can be divided into parametric and non-parametric models [63]. Parametric models explicitly determine parameterized expressions of independent variables (e.g., spectral bands) and the dependent variable of interest (e.g., AGB) assuming the probability distributions of the variables being assessed [63]. Multiple linear regression, a classic parametric model with normality assumption, was the most widely used method in previous AGB studies due to their simplicity and interpretability (e.g., [53,64,65]). Other parametric models, like non-linear regression (e.g., an exponential, power, or polynomial fitting function), were also applied for AGB estimations (e.g., [59,66,67]). Unlike parametric models, nonparametric models are distribution-free methods in which the predictor does not take a predetermined form but is constructed according to information derived from the data. Most machine learning models belongs to non-parametric, such as artificial neural network (ANN), random forest (RF), k-nearest neighbor (KNN), support vector machine (SVM), cubist (CB), classification and regression tree (CART), convolutional neural networks (CNN) and so on. Without the assumption of distribution, the non-parametric machine learning models are extremely flexible and capable of capturing the complex relationships between remote sensing variables and AGB, and widely applied in AGB estimation (e.g., [43,68,69,70,71,72,73]).

Ensemble learning, a branch of machine learning, is designed to learn tasks by constructing and then integrating multiple learners to produce a strong learner for improving accuracy [74,75]. There are three basic categories of ensemble learning: bagging, boosting, and stacking. RF and adaptive boosting (AdaBoost) algorithms are classic representatives of bagging and boosting algorithms, respectively. RF builds trees using subsamples and a random subset of predictors and can be very effective for estimating AGB due to its robustness to overfitting and noise in the training dataset [43,76,77]. Adaptive boosting is an iterative boosting algorithm that adaptively changes the distribution of the training set based on the performance of previous learners. Another boosting algorithm, called extreme gradient boosting (XGBoost), has been demonstrated to show great advantages in decreasing overestimation of low AGB values and underestimation of high AGB values for a forest type-based biomass estimation using continuous forest inventory data and Landsat 8 imagery [54]. Stacking, first proposed by Wolpert [78], is another method for combining multiple models but is less used than bagging and boosting. Unlike the RF algorithm that the base learner is homogeneous (e.g., regression tree), stacking are heterogeneous ensemble algorithms that could integrate diverse base learners to generate a stronger learner. The stacking algorithm was used to estimate canopy height in forestry (e.g., [79]), however, its potential has not been fully explored in AGB estimation.

Although the synergistic utilization of ALS and optical passive imagery was proved to improve AGB estimation [48], the synergistic approach (i.e., features) has not been fully investigated, especially for NSFs with complex structures. For example, is it more efficient to apply a novel feature extracted from passive imagery and LiDAR data (e.g., *COLI*1 and *COLI*2 in [48]) or directly combine all the features from the two data sources (like [61])? In addition, will ensemble learning algorithms improve the accuracy of AGB estimation for NSFs? Inspired by these questions, this study aimed at exploring the effects of different synergistic approaches of features and ensemble learning algorithms on AGB estimation of NSFs of northeastern China based on ALS and Landsat 8 OLI (Operational Land Imager) imagery. Specifically, the objectives of this study were (1) to investigate the effects of different data sources and classic machine learning algorithms on AGB estimation of a natural secondary forest; (2) to grope for a highly effective approach to combine ALS and Landsat 8 OLI imagery on AGB estimation of a natural secondary forest; (3) to explore the performances of ensemble learning algorithms in estimating AGB of a natural secondary forest; (4) to generate an accurate wall-to-wall AGB map of a natural secondary forest for future forest resources management.

## 2. Materials and Methods

### 2.1. Study Area

**Figure 1:**
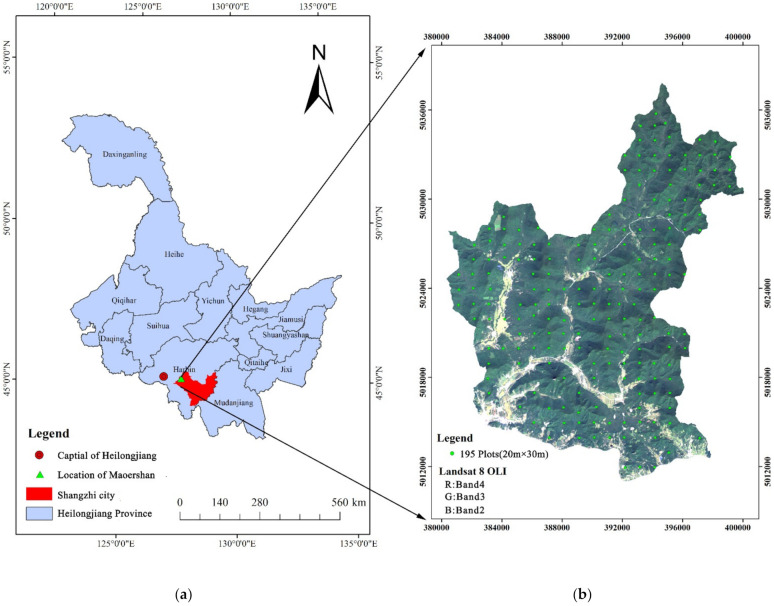
The location of study area: (**a**) The location of Maoershan Experimental Forest Farm within Heilongjiang Province; (**b**) the locations of 195 plots (20 m × 30 m) within Maoershan (Background: Landsat 8 OLI image).

The study area is located in Maoershan Experimental Forest Farm of Northeast Forestry University (NEFU), Shangzhi, Heilongjiang Province, China, ranging from 127°29′ to 127°44′ E and 45°14′ to 45°29′ (Figure 1). The landform of the forest farm belongs to a low mountain and hilly area. The terrain gradually rises from south to north, with an average elevation of 300 m. The highest mountain is Maoer Mountain, with an elevation of 805 m.

The total area of the forest farm is 26,496 ha, which belongs to a typical natural secondary forest in northeastern China. The vegetation in the Maoershan area is a part of Changbai plant flora, with the original zonal top-level community of Korean pine broad-leaved forest. Due to the destruction in the last century, the original vegetation has undergone reverse succession. It has formed a forest landscape in which natural secondary forests are dominated by precious broad-leaved forests, poplar and birch forests, oak forests, and so on, and plantations such as red pine and larch are inlaid. The main species include *Betula platyphylla*, *Quercus mongolica*, *Populus davidiana*, *Larix olgensis*, *Pinus sylvestris*, and *Pinus koraiensis*, etc. The average forest coverage rate is 95%, and the total stock is approximately 3.5 million m^3^.

### 2.2. Data Collection

#### 2.2.1. Remotely Sensed Data

The remotely sensed data utilized in this study include ALS data and Landsat 8 OLI imagery. ALS data were obtained in September 2015. It is a secondary product scanned by the LiDAR sensor (Riegl LMS-Q680i) carried by the LiCHY system of the Chinese Academy of Forestry. The maximum frequency of the laser pulse of the LiDAR sensor is 400 kHz, with a wavelength of 1550 nm, a scanning angle of ±30°, a sampling interval of 1 ns, and vertical accuracy of 0.15 m. The sidelap of this flight strip was designed to be greater than 60%, with an average point cloud density of 3.6 points·m^−2^.

To be consistent with ALS data in time, the Landsat 8 OLI imagery acquired on 13 September 2015 was applied in this study (downloaded from https://earthexplorer.usgs.gov/ (accessed on 1 September 2021)). The scene ID is LC81170282015256LGN01 (L1T-level product), with cloudiness of 1.35%, sun elevation angle of 45.28°, and sun azimuth angle of 154.91°. Seven multispectral bands (band1–band7) of 30 m nominal spatial resolution were utilized in this study. The radiometric resolution of the imagery is 12 bits and the swath width is 185 km × 185 km.

#### 2.2.2. Reference Data

The 195 fixed plots data of continuous forest resources inventory obtained in 2016 was applied as reference data in this study (see Figure 1b). The plot size was 20 m × 30 m and the center of each plot was correctly determined using a GPS (accuracy ±5 m). The diameter at breast height (DBH) of the trees larger than 5 cm and the tree species of each plot were recorded.

The AGB of individual trees was calculated using the species-specific allometric growth equations with DBH. In this study, the allometric growth models developed by [80,81] for the major species of trees and understory in northeastern China were employed to calculate the AGB of individual trees. The allometric growth equation was showed as Equation (1) and the parameters of major species of trees and understory were listed in Table 1.

(1) $$W=a\cdot D^{b}$$

where *W* represents aboveground biomass (kg), *D* represents DBH (cm), *a* and *b* are estimated parameters of different species in [80,81]. The AGB of the plot was the cumulative summation of the AGB of individual trees of each plot.

### 2.3. Methods

**Figure 2:**
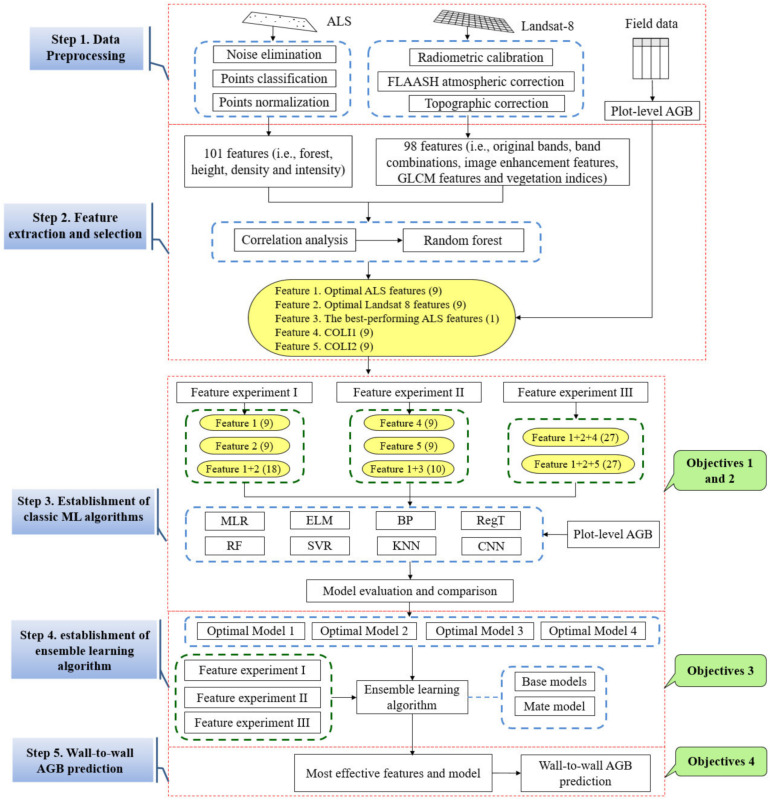
The flowchart of this study. Note: the number in parentheses represents feature number. Feature 1: optimal ALS features; Feature 2: optimal Landsat 8 features; Feature 3: the best performing ALS feature; Feature 4: all *COLI*1s; Feature 5: all *COLI*2s.

To investigate the effects of different synergistic approaches of features and ensemble learning algorithms on AGB estimation of NSFs, a five-step methodology with three experiments of features (Feature experiments I-III) was implemented in this study, including (1) data preprocessing, (2) feature extraction and selection, (3) establishment and evaluation of classic machine learning models, (4) establishment and evaluation of ensemble learning models, (5) wall-to-wall AGB prediction using the most effective algorithm and features. Feature experiment I was designed to explore the effects of features from different data sources (ALS, optical imagery, and combined data) on AGB estimation based on a variety of machine learning algorithms; Feature experiment II was designed to investigate how to efficiently combine the best-performing ALS feature (a unique feature) with several spectral features for AGB estimation, is it better to use novel extracted features or directly combine all the features?; Feature experiment III aims to compare the performance of combining all features for AGB estimation. The feature experiment design and logic of this study were shown in Table 2 and Figure 2, respectively.

#### 2.3.1. Preprocessing of Remotely Sensed Data

The preprocessing of the ALS data includes (1) noise elimination (such as air points, low points, and isolated points). The radius of a fitting plane and the multiples of standard deviation were set to 0.5 m and 1, respectively. The algorithm will automatically calculate the standard deviation of the surrounding fitting plane of a point. If the distance from this point to that plane is less than multiples of standard deviation, this point will be kept. (2) classification of ground and non-ground points. The ground points were classified by improved progressive triangulated irregular network densification (IPTD) filtering algorithm developed in [82]. The maximum building size and maximum terrain angle were set to 20 m and 88°, respectively. (3) normalization of point clouds. A digital terrain model (DTM) with a resolution of 0.5m was generated based on ground points using the inverse distance weighted (IDW) interpolation method. The power of the distance between sampling points and an unknown point was set to 2, and the smallest number of points used for interpolation was 12. Then, the point clouds were normalized by subtracting the DTM value from the elevation of all points. The preprocessing of the ALS data was implemented using LiDAR 360 V3.2 of GreenValley International.

Preprocessing of the Landsat 8 OLI imagery including radiometric calibration, atmospheric correction, and topographic correction was implemented using ENVI 5.3 software. The Fast Line-of-sight Atmospheric Analysis of Spectral Hypercube (FLAASH) radiative transfer model was implemented for atmospheric correction and conversion to surface reflectance in the EVNI environment. The topographic correction was conducted with the well-known Sun Canopy Sensor + C correction (SCS + C) approach using the extension tool of “Topographic Correction_V5.3_4_S1”. The SCS + C correction approach reduces overcorrection and is an effective topographic correction method in forested and mountainous terrain [83,84]. The SCS + C topographic correction model can be expressed by Equation (2).

(2) $$L_{t}=L\cdot \left(\frac{cos\theta \cdot cos\alpha +C}{cosi+C}\right)$$

where $L_{t}$ is the corrected pixel radiance value of the image; *L* is the uncorrected pixel radiance value of the image; i  is the incidence angles on a horizontal surface; *θ* is the solar zenith angle; α is the slope angle; C is the semi-empirical parameter. DTM generated from ALS data was applied for topographic correction in this study.

#### 2.3.2. Feature Extraction and Selection

- Feature Extraction

Four categories of 101 features related to forest, height, density, and intensity features were derived from normalized ALS point cloud data. Forest features include canopy cover, leaf area index (LAI), and gap fraction. Canopy cover refers to the proportion of the forest floor covered by the vertical projection of the tree crowns [85]. LAI is one of the most significant variables for representing canopy structure, with the definition of half the total foliage area per unit ground surface area [86]. The gap fraction can be calculated by the ratio of the number of ground points whose elevation is lower than the height threshold (i.e., 2 m in this study) and the total return number. All 101 ALS features, including three forest metrics, 46 elevation metrics, 10 density metrics, and 42 intensity metrics were extracted using LiDAR 360 V3.2 of GreenValley International. The feature details were listed in Table A1 of Appendix A.

A variety of features could be derived from optical imagery. According to previous studies (e.g., [48,54,73,87]), band combinations, vegetation indices, textures (e.g., gray-level co-occurrence matrix (GLCM)) of each band, and image transformations (e.g., principal component analysis, tasseled cap, minimum noise fraction) were extracted as potential predictors for AGB modeling. Therefore, 98 features were selected or extracted from Landsat 8 imagery in this study, including seven original bands (band 1–7), ten band combinations, ten image enhancement features (i.e., three principal components, three tasseled-cap features, and four minimum noise fractions), 56 GLCM features, and 15 vegetation indices. The details of the 98 features derived from Landsat 8 were listed in Table A2 of Appendix A.

- Feature Selection

To avoid the “curse of dimensionality”, it is a prerequisite to select the most effective feature for AGB estimation. In this study, the two-step feature selection procedure is implemented, including (1) preliminary selection using Pearson correlation coefficient; and (2) further selection based on variable importance measure using random forest. For the first step, Pearson correlation coefficients of each feature and AGB were calculated and the features with *p*-value less than 0.05 that significantly correlated with AGB were selected. Then, the selected features were ranked according to variable important measures calculated with random forest. Due to the randomness, the ranking procedure was implemented 10 times to find out the most stable set of features with high ranking.

The two-step feature selection was implemented for ALS and Landsat 8 data, respectively, to select two sets of best-performing features. Among the selected ALS features, the best-performing ALS variable was determined by establishing and evaluating the univariate models of each ALS feature and AGB. The feature selection procedure was implemented using R version 4.0.4 (https://www.r-project.org/ (accessed on 1 September 2021)). 

According to [48], two types of indices (*COLI*1 and *COLI*2) incorporating optical imagery and ALS information were established using the best-performing LiDAR variable with each optical spectral vegetation index. The best-performing LiDAR variable was determined by the univariate model of AGB and the LiDAR variable with the highest R^2^. The best-performing spectral features of Landsat 8 were selected by the two-step feature selection procedure described above. Then, the generation of *COLI*1 and *COLI*2 based on the best-performing LiDAR variable (only one feature) and the best-performing Landsat 8 features (could be several features) included both feature selection and extraction procedures. For convenience, we still used the notation of [48] but adjusted the equations as follows.

(3) $$COLI1={SF}_{i}\times BLV$$

(4) $$COLI2={SF}_{i}\_BLV=\frac{\left(BLV-SF_{i}\right)}{\left(BLV+SF_{i}\right)}$$

where *BLV* is the best-performing LiDAR variable (only one feature), *SF_i_* is a set of best-performing features derived from Landsat 8 imagery (several features). Thus, the number of *COLI*1 or *COLI*2 is identical to the number of best-performing spectral features (*SF_i_*).

#### 2.3.3. Classic Machine Learning Algorithms

In this study, seven classic machine learning algorithms were conducted to estimate the AGB of NSFs, including extreme learning machine (ELM), backpropagation (BP) neural network, regression tree (RegT), RF, support vector regression (SVR), KNN, and CNN. Traditional multiple linear regression (MLR) was applied as a baseline for model comparison.

- ELM

ELM is a class of machine learning methods built on the feedforward neuron network (FNN) for supervised and unsupervised learning problems [88]. ELM is an improvement of FNN and its backpropagation algorithm, which is characterized by random or artificially given weights of the nodes in the hidden layer and does not need to be updated. Compared to single-layer perceptron and SVM, ELM is considered to have possible advantages in terms of learning rate and generalization ability [88].

- BP

BP neural network, proposed by Rumelhart et al. in 1986 [89], is a multilayer feedforward network trained by error backpropagation algorithm and is one of the most widely used neural network models [90]. Its learning rule is to use the fastest descent method to continuously adjust the weights and thresholds of the network by backpropagation to minimize the sum of squared errors of the network. According to error and trials, the BP algorithm was implemented with epochs of 1000 in this study.

- RegT and RF

A regression tree is a basic method built on the principle of minimizing the loss function for a regression problem. The major advantage of the regression tree is the readability of the model and fast computational speed, which make it particularly suitable for integrated learning, such as random forests. RF, proposed by Leo Breiman [76], is based on multiple regression trees, which is capable of capturing the complicated relationship between a response and a set of explanatory variables with the following advantages: robustness to reduce over-fitting, ability to determine variable importance, higher accuracy, fewer parameters that need to be tuned, lower sensitivity to the tuning of the parameters, fast training speed, and anti-noise property. The number of regression trees and the random state of the RF algorithm were set to 1000 and 10, respectively, in this study.

- SVR

SVM is a class of generalized linear algorithms that performs the classification of data in a supervised learning manner, where the decision boundary is the hyperplane of maximum margins solved for the learned samples. SVR is a transformation of SVM designed for regression problems and can perform nonlinear problems by kernel method. Linear kernel and penalty factor of 1 were applied for SVR in this study.

- KNN

The KNN method is a multivariate nonparametric algorithm that uses a set of predictors (Xs) to match each target pixel to a number (K) of most similar (nearest neighbors) reference pixels for which values of response variables (Y) are known. The number of nearest neighbors was set to 5 and uniform weights were utilized in this study.

- CNN

CNN, firstly developed in 1995 for the classification of handwritten images [91], is one of the most representative algorithms of deep learning. CNN interprets spatial data by scanning it using a series of trainable moving windows and has the capability of representation learning in a translation-invariant manner according to its hierarchical structure. In this study, the CNN model had a simple structure with an input layer, a hidden layer, and an output layer, and was implemented using an epoch of 1000 and a batch size of 30.

#### 2.3.4. Ensemble Learning Algorithms

**Figure 3:**
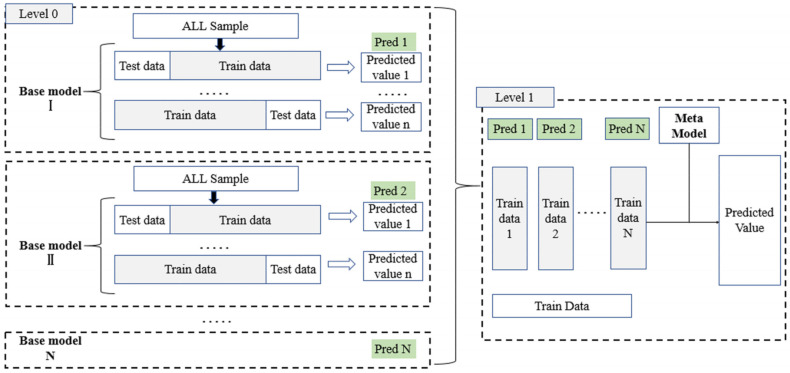
Flowchart of stacked generalization (SG) algorithm in this study. Note: The number of the base model (N) was set to four in this study and 195 iterations were running within each model because of the leave-one-out cross-validation of 195 sample plots.

Stacked generalization (SG) which is a layered ensemble learning algorithm [92] was applied in this study. There are two layers designed in the SG algorithm here, including basic models and meta models. The input of the base model is the original training set and the output of the base model is applied as the training set for meta model [93]. The meta model could be a single model or an ensemble model [93,94], like RF. To obtain a better performance of SG, the base models should be accurate and different as much as possible. Thus, the four best-performing machine learning algorithms described in Section 2.3.3 were selected for the base models according to leave-one-out cross-validation and meta models for establishing SG algorithms in this study, which resulted in four SG algorithms. The flowchart of the SG algorithm in this study was presented in Figure 3. 

#### 2.3.5. Model Evaluation

This study adopted a leave-one-out cross-validation method to evaluate the model accuracy. Since 195 sample plots were used in this study, the training and testing data were 194 plots and 1 plot, respectively; and 195 iterations were run for each model. Due to the problems of coefficient of linear determination (*R*^2^) for nonlinear models [95], we avoid applying *R*^2^ of machine learning models established by selected features and AGB. However, *R*^2^ of actual and predicted AGB could be used as an indicator since the relationship of actual and predicted AGB can be described by a simple linear model. Therefore, six indices were applied for model evaluation, including *R*^2^ of actual and predicted AGB, root mean squared error (RMSE), relative root mean squared error (rRMSE), mean absolute error (MAE), mean absolute percentage error (MAPE), and precision measure (PM). The equations were shown as follows:

(5) $$R^{2}=\frac{\sum_{i=1}^{n}{\left({\hat{y}}_{i}-\bar{y}\right)}^{2}}{\sum_{i=1}^{n}{\left(y_{i}-\bar{y}\right)}^{2}}$$

(6) $$RMSE=\sqrt{\frac{1}{n}\overset{n}{\underset{i=1}{\sum }}{\left(y_{i}-\hat{y}\right)}^{2}}$$

(7) $$rRMSE=\frac{\sqrt{\frac{1}{n}\sum_{i=1}^{n}{\left(y_{i}-\hat{y}\right)}^{2}}}{\bar{y}}$$

(8) $$MAE=\frac{1}{n}\overset{n}{\underset{i=1}{\sum }}\left|y_{i}-{\hat{y}}_{i}\right|$$

(9) $$MAPE=\frac{1}{n}\overset{n}{\underset{i=1}{\sum }}\frac{\left|y_{i}-{\hat{y}}_{i}\right|}{y_{i}}\times 100\%$$

(10) $$PM=\frac{\sum_{i=1}^{n}{\left(y_{i}-{\hat{y}}_{i}\right)}^{2}}{\sum_{i=1}^{n}{\left(y_{i}-\bar{y}\right)}^{2}}$$

where *n* represents the number of observation samples, *y_i_* represents the actual AGB of the *i*th plot, ${\hat{y}}_{i}$ represents the predicted AGB of the *i*th plot, and $\bar{y}$ represents the mean of the actual AGB. All the model fitting and evaluation procedures in this study were implemented by python 3.7 (https://www.python.org/downloads/ (accessed on 1 September 2021)), TensorFlow 2.2 (https://tensorflow.google.cn/ (accessed on 1 September 2021)) and sklearn (https://scikit-learn.org/stable/ (accessed on 1 September 2021)).

## 3. Results

### 3.1. Feature Selection

Due to a large number of extracted features (199 features in total), two-step feature selection was implemented in this study, including preliminary selection using Pearson correlation coefficient; and further selection based on variable importance measure using random forest. Finally, nine ALS features were selected and sorted from highest to lowest variable importance as follows: elev_mean, int_AII_5th, elev_cv, density_7th, int_max, int_AII_40th, int_per_60th, int_per_80th, and int_AII_50th; nine features extracted from Landsat 8 were selected and ranked in descending order of variable importance: MVI5, B1, B76, B65, B53, Entr_B5, B2, ND563, and MVI7. The selected features and their descriptions were listed in Table 3.

To grope for the best-performing ALS feature, simple linear regressions were established to model the relationship between AGB and each ALS feature. The result of univariate models showed that the elevation mean outperformed other ALS features due to higher *R*^2^ and lower *RMSE*, *rRMSE*, *MAE*, *MAPE*, and *PM* (Table 4). Thus, elevation mean was selected as the best-performing ALS feature to generate *COLI*1 and *COLI*2 using Equations (3) and (4).

### 3.2. Performance of Classic Machine Learning Algorithms

#### 3.2.1. Experiment I

The goal of feature experiment I was to explore the effects of features from different data sources (optical imagery, ALS, and combined data) on AGB estimation based on seven classic machine learning algorithms, including ELM, BP, RegT, RF, SVR, KNN, and CNN. MLR was implemented as a baseline for model comparison. Table 5 shows the performance of the eight models using the three sets of features designed in Experiment I. 

In general, the optimal ALS features (Feature 1) performed significantly better than the optimal Landsat 8 features (Feature 2) for AGB estimation, no matter of algorithms; the combination of the optimal ALS and Landsat 8 features (Feature 1 + 2) performed differently for various algorithms. For each data source, the accuracy of CNN was greatly higher than that of other algorithms, especially for applying both ALS and Landsat 8 features (*R*^2^ = 0.97, *RMSE* = 12.6, *rRMSE* = 0.08, *MAE* = 6.43, *MAPE* = 4.02, *PM* = 0.13). However, it is worth mentioning that the accuracies of other algorithms (except CNN) based on two data sources (Feature 1 + 2) were not significantly improved compared with those based on optimal ALS features (Feature 1), which suggested that the accuracy of AGB estimation not only depends on data sources but also different algorithms. Some algorithms (like RF and SVR) could provide very similar accuracy using both optimal ALS and Landsat 8 features to that using only optimal ALS features, making it meaningless to involve optical imagery. Thus, ALS data are of significance to AGB estimation. 

#### 3.2.2. Experiment II

After determining the best-performing ALS feature (i.e., elevation mean), we designed feature experiment II to investigate how to efficiently combine the unique feature with the optimal Landsat 8 features for AGB estimation. Is it better to utilize a novel feature extracted from elevation mean and optimal Landsat 8 features (i.e., *COLI*1 and *COLI*2) or directly combine all the features? A similar feature size in experiment II (i.e., 9 or 10) could avoid the unfair comparison due to the big difference in feature number. Table 6 presented the accuracy assessment of classic machine learning algorithms with three sets of features designed in experiment II. The results showed that the addition of elevation mean significantly improves the accuracies of AGB estimation compared to those using optical features only (Feature 2), no matter how to add it. The models except CNN had very similar performances in AGB estimation for the three feature combinations in experiment II. CNN still showed great advantages like Experiment I, especially for the case of simply combining the optimal Landsat 8 features and elevation mean together (Feature 2 + 3) with the accuracy of *R*^2^ = 0.88, *RMSE* = 24.48, *rRMSE* = 0.16, *MAE* = 10.19, *MAPE* = 7.23, and *PM* = 0.24, followed by the case of all *COLI*2 (Feature 5), and then the case of all *COLI*1 (Feature 4). Thus, it seemed unnecessary to generate the new features (i.e., *COLI*1 or *COLI*2) when CNN was applied for AGB estimation based on the optimal Landsat 8 features and the best-performing ALS feature for NSFs.

#### 3.2.3. Experiment III

To investigate the effect of combing optimal ALS and Landsat 8 features and two types of novel features (*COLI*1 or *COLI*2) using classic machine learning algorithms, experiment III was implemented (Table 7). Comparing to the result of applying optimal ALS and Landsat 8 features (Feature 1 + 2) in Table 5, the additions of the novel features, no matter *COLI*1 or *COLI*2, slightly improved the accuracies of most models, like MLR, BP, RegT, RF, and KNN. In addition, the accuracies of all models except RF using optimal ALS and Landsat 8 features and all *COLI*2 (Feature 1 + 2 + 5) were slightly improved compared to those using optimal ALS and Landsat 8 features and all *COLI*1 (Feature 1 + 2 + 4), indicating *COLI*2 were more efficient than *COLI*1 for AGB estimation of NSFs. CNN was still much superior to other algorithms and reached the highest accuracies (*R*^2^ = 0.99, *RMSE* = 6.85, *rRMSE* = 0.04, *MAE* = 2.95, *MAPE* = 1.02, *PM* = 0.03) when optimal ALS and Landsat 8 features and all *COLI*2 (Feature 1 + 2 + 5) was applied.

### 3.3. Performance of Ensemble Learning Algorithms

#### 3.3.1. Experiment I

To explore the performances of ensemble learning algorithms in estimating AGB based on different feature combinations, Experiment I, II, and III were also implemented using the designed SG algorithms. According to the results of classic machine learning algorithms (Table 5, Table 6 and Table 7), four best-performing models, that is, RF, SVR, KNN, and CNN, were selected as base models for the SG algorithm. The predictions of base models were applied as the input of the meta model of the SG algorithms, which were also RF, SVR, KNN, and CNN. Thus, there were four SG algorithms due to four meta models, including SG(RF), SG(SVR), SG(KNN), and SG(CNN). Table 8 presented the accuracy assessment of ensemble learning algorithms with three sets of features designed in experiment I. Comparing to the results of base models (Table 5), the SG algorithms greatly improved the accuracy of AGB estimation using the optimal Landsat 8 features (Feature 2) and the combined optimal features (Feature 1 + 2). However, for the case of optimal ALS features (Feature 1), the SG algorithms had slightly lower accuracies than those of base models, except CNN. In general, CNN still performed best as a meta model of SG algorithm, followed by SG algorithm with SVR meta model, and finally with RF meta model as well as KNN model. Although CNN was still an outstanding meta model for all the cases, it was worth noting that the drastic improvements of accuracies brought by SG(SVR), SG(RF), and SG(KNN) compared with their corresponding base model, especially for the Feature 2 and Feature 1 + 2. For example, *R*^2^ of SG(SVR), SG(RF), and SG(KNN) increased approximately 30%–40% and 60%–70% for Feature 2 and Feature 1 + 2, respectively; alternatively, *R*^2^ of SG(CNN) only increased 49% and 0% for Feature 2 and Feature 1 + 2, respectively. Other indices (*RMSE*, *rRMSE*, *MAE*, *MAPE*, and *PM*) had similar trends, but in the opposite direction. Thus, it had more room for improvement to apply the SG algorithms for relatively weaker learners (like SVR, RF, and KNN) than strong deep learning learners (like CNN).

#### 3.3.2. Experiment II

Feature experiment II was also implemented to investigate how to integrate elevation mean and the optimal Landsat 8 features for AGB estimation based on ensemble learning algorithms (Table 9). It showed that the SG algorithms greatly improved the accuracies for all the cases except the SG(CNN) for Feature 5 and Feature 2 + 3, comparing to the accuracies using the corresponding base model (Table 6). When SG algorithms were utilized, the trend that the simple combination of optimal Landsat 8 features and elevation mean (Feature 2 + 3) performed best, followed by all *COLI*2 (Feature 5), and finally all *COLI*1 (Feature 4) was much more obvious than that using classic machine learning algorithms (Table 6 vs. Table 9). The advantage of applying deep learning algorithm CNN as meta model decreased with the dramatic increase in the accuracies of the other three algorithms (i.e., RF, SVR, and KNN), especially for Feature 5 and Feature 2 + 3. In other words, when the feature set of all *COLI*2 or the feature set of optimal Landsat 8 features and elevation mean was applied for AGB estimation, SG(RF), SG(SVR), and SG(KNN) had comparable accuracies to SG(CNN).

#### 3.3.3. Experiment III

The effect of combing optimal ALS and Landsat 8 features and two types of novel features (*COLI*1 or *COLI*2) on AGB estimation using ensemble algorithms was investigated with experiment III (Table 10). Unlike classic machine learning algorithms, the addition of *COLI*1 in ensemble algorithms did not improve the accuracies of AGB estimation, compared to the result of applying optimal ALS and Landsat 8 features (Feature 1 + 2) in Table 8. The SG(SVR) or SG(KNN) with the addition of *COLI*1 even lower R^2^ by about 10%–20% than SG(SVR) or SG(KNN) with only Feature 1 + 2 (Table 8). However, the addition of *COLI*2 in ensemble algorithms slightly increased the accuracies of most models except SG(KNN), even though SG algorithms with Feature 1 + 2 had already performed well (Table 8). In general, the SG algorithms with optimal ALS and Landsat 8 features and all *COLI*2 (Feature 1 + 2 + 5) had more stable accuracies than that with optimal ALS and Landsat 8 features and all *COLI*1 (Feature 1 + 2 + 4), no matter which meta model was used, indicating *COLI*2 were more efficient than *COLI*1 for AGB estimation of NSFs. It is still the SG model with CNN meta model that has the highest accuracy (*R*^2^ = 0.99, *RMSE* = 2.02, *rRMSE* = 0.01, *MAE* = 0.87, *MAPE* = 0.73, *PM* = 0.02) when optimal ALS and Landsat 8 features and all *COLI*2 (Feature 1 + 2 + 5) was applied.

In addition, the ensemble algorithms greatly improved the accuracies of the corresponding features and base model (Table 10 vs. Table 7). For example, if the combination of optimal ALS and Landsat 8 features and all *COLI*1 (Feature 1 + 2 + 4) was utilized, the *R*^2^ of SG(RF) increased more than 60% compared with that of the RF model; *RMSE*, *rRMSE*, *MAE*, *MAPE* and *PM* of SG(RF) decreased by 75%, 73%, 71%, 76%, and 81%, respectively, compared with those of the RF model. Although the CNN base model had already achieved high accuracy, especially when applying the combination of optimal ALS and Landsat 8 features and all *COLI*2 (Feature 1 + 2 + 5 in Table 7), the SG(CNN) still decreased the group of *RMSE*, *rRMSE*, and *MAE* and the group of *MAPE* and *PM* by about 70% and 30%, respectively.

### 3.4. Wall-to-Wall AGB Predictions

**Figure 4:**
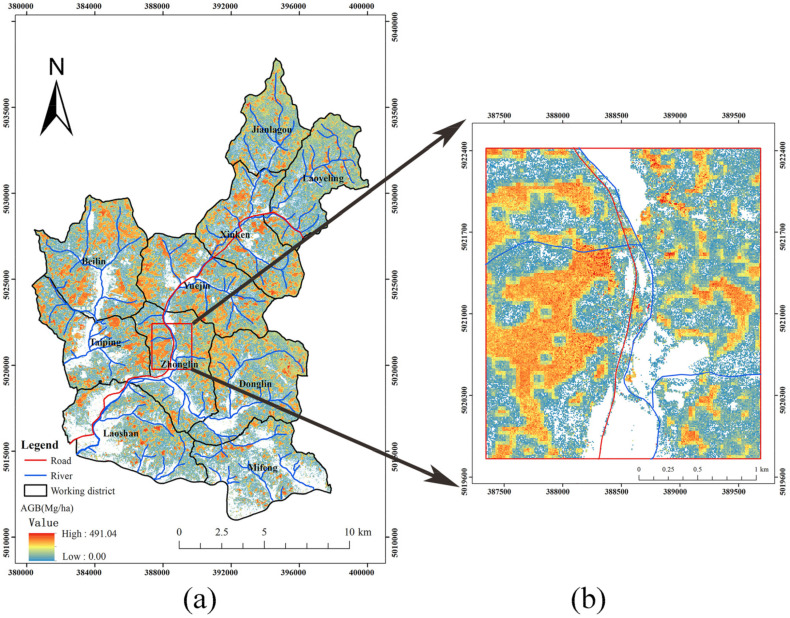
(**a**) The wall-to-wall AGB prediction of the entire study area estimated by the CNN model with optimal ALS features, optimal Landsat 8 features, and all *COLI*2 (Feature 1 + 2 + 5); (**b**) Spatial distribution of AGB for a partial area in Zhonglin working district.

Based on the above results and algorithm efficiency, CNN and the feature set of optimal ALS and Landsat 8 and all *COLI*2 (Feature 1 + 2 + 5) were selected for a wall-to-wall AGB prediction of the entire Maorshan Experimental Forest Farm of NEFU (Figure 4). The predicted AGB varied from 0 to 491.04 Mg/ha, with a mean value of 59.9 Mg/ha and a standard deviation of 48.69 Mg/ha. The area with AGB of 0 or low values was located along rivers, roads, or residential regions, whereas the area with high AGB values was located in the center part (e.g., Zhonglin, Yuejin, Beiling, Donglin, and Xinken working districts) of Maorshan (Figure 4a). However, the embedded pattern of high and low AGB values was obvious for most of the study area, as the enlarged area in Zhonglin working district (Figure 4b).

**Figure 5:**
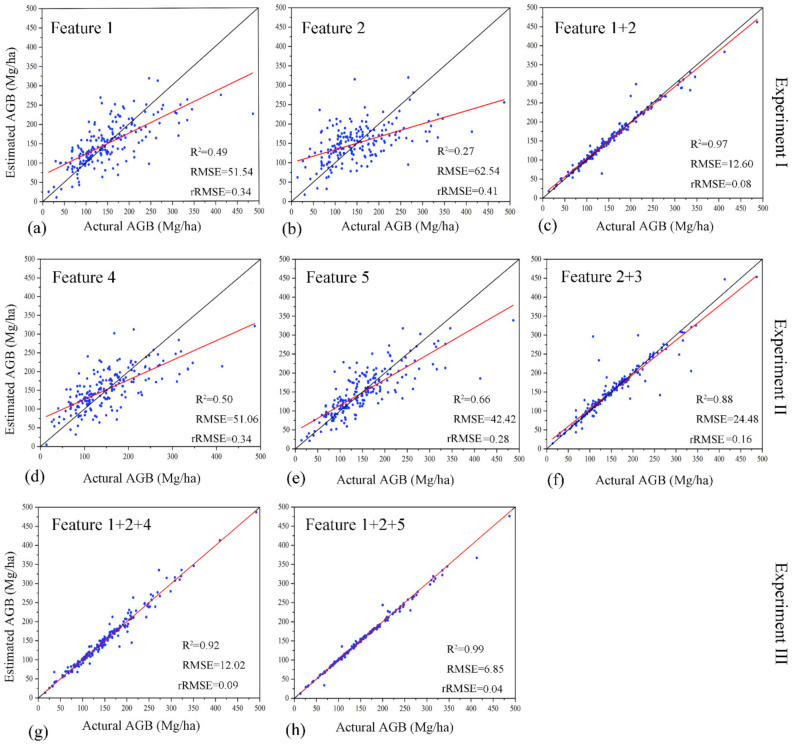
The relationship of actual and estimated AGB (Mg/ha) of 195 plots using CNN algorithm based on (**a**) Feature 1: optimal ALS features; (**b**) Feature 2: Optimal Landsat 8 features; (**c**) Feature 1 + 2: Optimal ALS and Landsat 8 features; (**d**) Feature 4: All *COLI*1; (**e**) Feature 5: All *COLI*2; (**f**) Feature 2 + 3: Optimal Landsat 8 features and the best performing ALS feature; (**g**) Feature 1 + 2 + 4: Optimal ALS features, optimal Landsat 8 features, and all *COLI*1; (**h**) Feature 1 + 2 + 5: Optimal ALS features, optimal Landsat 8 features, and all *COLI*2. Note: The red and black lines represent the fitted regression lines and the line of 45°, respectively.

Figure 5 showed the relationship of actual and estimated AGB (Mg/ha) of 195 plots using the CNN algorithm based on different feature sets. For experiment I, it was better to apply ALS than Landsat 8 to predict AGB if only one data source had to be used, which indicated the vertical forest structure was more vital than spectral information for AGB estimation of NSFs. The synergism of optical imagery and ALS markedly increased the accuracy of a single data source (Figure 5c vs. Figure 5a or Figure 5b) since it could effectively alleviate the underestimation of high AGB values. Even only one ALS feature (i.e., elevation mean) was added to the Landsat 8 features (Experiment II), the improvement was obvious and significant. However, it was unnecessary to generate novel features like *COLI*1 or *COLI*2 using the optimal Landsat 8 and elevation mean. It was in evidence that the performance of directly combining them was much better than that of new features (Figure 5f vs. Figure 5d) or Figure 5e), but worse than that of all optimal ALS and Landsat 8 features (Figure 5f vs. Figure 5c) due to the smaller number of features (i.e., 10 vs. 18). The effectiveness of *COLI*1 was very limited because Feature 1 + 2 provided a comparable result to Feature 1 + 2 + 4 (Figure 5c vs. Figure 5g). It is the most efficient to combine all optimal ALS, Landsat 8, and *COLI*2 features, especially for estimating high AGB values (Figure 5h).

## 4. Discussion

### 4.1. AGB Estimation Using Different Features

The differences in features are responses to the characteristics of different data sources. In this study, we extracted a variety of features and investigated the effects of different synergistic approaches of features derived from ALS and Landsat 8 OLI imagery on AGB estimation of NSFs of northeastern China. For ALS data, besides elevation features, density- (e.g., density_metrics7) and intensity-related (e.g., int_AII_5th, int_max, int_AII_40th, int_per_60th, int_per_80th, and int_AII_50th) metrics also had great potentials in AGB estimation; for Landsat 8 imagery, band combinations and texture are more efficient than vegetation indices, especially MVI5 (i.e., the band combination of band 5, 4 and 2). Unfortunately, some traditional vegetation indices that commonly applied in previous studies [48], for example, the normalized difference vegetation index (NDVI), enhanced vegetation index (EVI), atmospherically resistant vegetation index (ARVI), soil adjusted vegetation index (SAVI), etc., were excluded due to the low correlations with AGB. Only one vegetation index (i.e., ND563) was selected. It might be because that study area is a natural secondary forest with high canopy density which could easily result in the saturation (insensitivity to AGB) of the traditional vegetation indices, which was also confirmed in [96,97]. The low accuracies (e.g., *R*^2^ < 0.3) of AGB estimations using the optimal Landsat 8 features (Feature 2), no matter of algorithms, indicating the difficulties of AGB estimation of NSFs as well. Due to the vegetation characteristics, near-infrared and shortwave infrared bands (i.e., band 5, 6, and 7) were more related to AGB estimation than other bands. 

Similar to previous studies [48,52,98], it was beneficial to combine ALS data and optical imagery, even only combining one significant feature derived from ALS (like elevation mean). The synergistic method of extracting novel features (i.e., *COLI*1 and *COLI*2) using optimal Landsat 8 features and the best-performing ALS feature (i.e., elevation mean) yielded higher accuracy of AGB estimation than either optical-only or ALS-only features when the same model was implemented. From experiment II and III, it showed that *COLI*2 had more advantages than *COLI*1 in AGB estimations of NSFs, which is different from [48] due to different forest types (NSFs of northeastern China vs. mixed forests of southern China). However, it is surprised to find out that the novel extracted features (*COLI*1 and *COLI*2) were not efficient in improving the accuracy compared to the simple combination of the untransformed features (optimal Landsat 8 features + BLV), which indicated the great convenience and effectiveness brought by just adding the best-performing ALS feature (i.e., elevation mean) to the original set of Landsat 8 features for AGB estimation of NSFs. The number of features was also a vital factor to influence the AGB accuracy. To make sure a fair comparison of synergistic approaches of features, we keep the number of features consistent as much as possible within each experiment. It is a trend that the accuracy of AGB estimation raises with the increase in the number of involved features under the same conditions (e.g., algorithms). Thus, it was not surprising that the combination with 27 features (i.e., Feature 1 + 2 + 4 or Feature 1 + 2 + 5) in experiment III provided the best performances in this study, from a feature size perspective. 

### 4.2. AGB Estimation Using Machine Learning Algorithms

The effect of classic machine learning and ensemble learning algorithms on AGB estimation using different features was explored in this study. The RF algorithm that is one of the most commonly used algorithms in forestry only provided very modest accuracy in this study since it constantly overfits the data, often with poorer predictions [33]. CNN, a deep learning algorithm firstly developed in 1995 for the classification of handwritten images [91], showed absolute advantages compared with other classic algorithms (e.g., ELM, BP, RF, KNN, SVR, etc.). As a representative of deep learning algorithms that is a branch of machine learning, a large and deep CNN (consisting of many-layered convolutions) was further developed in 2012 and achieved a winning top-5 test error rate of 15.3% in the ImageNet ILSVRC-2012 competition [99]. In recent years, the CNN model has been increasingly applied in forestry, for example, for the prediction of forest inventory parameters and identification of different tree species [100,101]. CNN interprets spatial data by scanning it using a series of trainable moving windows and sufficiently complex artificial neural networks and does not require human-derived feature selection in essence [100]. However, to make sure a fair comparison of different models, we keep the feature selection procedure consistent for all models. It means that the CNN model was applied for two-dimensional data of AGB and a set of human-derived features instead of a three-dimensional image. Although the CNN model lost the advantage of automatically extracting and selecting features, it is still sensitive to changes in features and significantly superior to other models (e.g., ELM, BP, RF, KNN, SVR, etc.).

The SG algorithms, a kind of ensemble learning algorithms, applied heterogeneous ensemble methods with different base models and greatly improved the AGB estimation accuracy in this study. RF, KNN, SVR, and CNN were selected as base models since SG algorithms could take advantage of the good and stable predictions from base models. The good prediction of the CNN base model successfully made the accuracy of the SG algorithms improved and stable no matter of meta-models, which indicated that SG has a stronger generalization ability than base models. In other words, it is more beneficial for weaker learners (e.g., RF, KNN, and SVR) to become stronger learners using SG algorithms than strong learners (e.g., CNN). 

However, although the SG algorithm is superior to its corresponding base model, we still recommend employing the CNN model for AGB estimation in practice due to its comparable accuracy and good efficiency. Table 11 summarized the efficiency (i.e., runtime) of all the algorithms with the combination of the optimal ALS and Landsat 8 features, and all COLI2 (Feature 1 + 2 + 5) for AGB estimation of 195 plots on a computer with AMD RX3700x + 16GB + GTX960 4GB. It showed that the runtime of ensemble algorithms (i.e., SG(RF), SG(KNN), SG(SVR), SG(CNN)) was dramatically augmented compared with their corresponding base model (i.e., RF, KNN, SVR, CNN). For example, the efficiency of SG(CNN) is only half of that of the CNN model. Other SG algorithms (i.e., SG(RF), SG(KNN), SG(SVR)) raised the runtime of the corresponding algorithm (i.e., RF, KNN, SVR) even more. The CNN model had the longest runtime but yield the highest accuracy (see Table 5, Table 6 and Table 7) among classic machine learning algorithms due to the most complex structure. Thus, to balance the workload and accuracy, the wall-to-wall AGB prediction map was generated using the CNN model with the combination of the optimal ALS and Landsat 8 features, and all COLI2 (Feature 1 + 2 + 5) in this study.

### 4.3. Comparison of Estimated Forest AGB and Current Publications

From the AGB accuracy perspective, the highest accuracy (*R*^2^ = 0.99, *RMSE* = 2.02, *rRMSE* = 0.01, *MAE* = 0.87, *MAPE* = 0.73, *PM* = 0.02) was yielded by SG(CNN) algorithm with the combination of the optimal ALS and Landsat 8 features and all *COLI*2 (Feature 1 + 2 + 5) in this study, which was better than other similar AGB studies that applied both LiDAR and optical imagery (e.g., [48,61,69,98,102]). Besides features and algorithms, the high accuracy of this study also benefited from the case of a local study with a relatively small area. It tends to decrease the accuracy for national and global scales. For example, Su et al. [69] provided the *R*^2^ of 0.75 and the RMSE of 42.39 Mg/ha for the AGB estimation of China based on ICESat GLAS laser altimetry data, MODIS, and forest inventory data. Yang et al. [103] produced a global forest AGB map with the *R*^2^ of 0.90 and the *RMSE* of 35.87 Mg/ha using gradient augmented regression trees algorithm based on multiple data sources (e.g., LiDAR-derived forest AGB datasets, field measurements, high-level products from optical satellite imagery, etc.).

**Figure 6:**
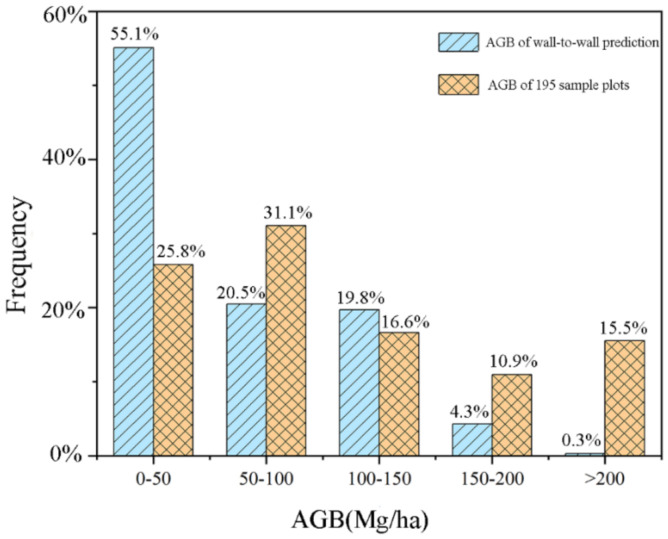
The distributions of AGB values of wall-to-wall prediction map (blue bars with one slash) and 195 sample plots (orange bars with double slashes).

Further, we dig into the predicted AGB values of the wall-to-wall map of the entire Maorshan and compared the distributions of AGB values of the wall-to-wall prediction map and 195 sample plots (Figure 6). Although the spatial distribution of AGB values of the wall-to-wall prediction map seemed to be reasonable (Figure 4), it showed that there was still a big difference between the two distributions, especially for the ranges of 0–50 Mg/ha and >200 Mg/ha (Figure 6), indicating the underestimation of high AGB values and overestimation of low AGB values. It suggested that the data saturation in Landsat imagery was not fully eliminated in this study of natural secondary forests. For Heilongjiang province, the average forest AGB density estimated by [69,104] was 81 Mg/ha and 85 Mg/ha, respectively (using a ratio of 50% for the conversion from forest AGB to AGB carbon stock); for the entire northeastern China, the average forest AGB density estimated by [57,105] was 83.50 Mg/ha and 89.30 Mg/ha, respectively. All these values were significantly higher than the average AGB of 59.9 Mg/ha in this study. The first reason for that could be the different study area: the area of either Heilongjiang province or northeastern China is much larger than Maorshan Experimental Forest Farm and includes the areas with high AGB values, such as Daxing’an Mountains, Xiaoxing’an Mountain, or Changbai Mountains, which results in a higher average AGB value. The second reason could be that the data saturation in this study greatly causes the relatively low average AGB, although the range of predicted AGB (0–491.04 Mg/ha) is reasonable. Thus, how to eliminate data saturation and quantitatively determine saturation for NSFs still need further investigation.

### 4.4. Limitations and Recommendations

The AGB retrievals with high accuracy from remotely sensed data is not an easy task. Every procedure or factor could greatly influence the accuracy, including data sources, feature extraction and selection, estimation models, and model evaluation, and so on. Although high accuracies of AGB estimation were yielded by the CNN and SG(CNN) models based on the combination of the optimal ALS and Landsat 8 features and all *COLI*2 (Feature 1 + 2 + 5), there were still limitations in this study. First, in this study, we only tested the features (*COLI*1, *COLI*2) proposed by [48] and compared them with the direct combination of these original features that generated them for the AGB estimation of NSFs. It is possible to find a more effective approach to combine ALS and Landsat 8 imagery than *COLI*s for NSFs. Thus, it is still valuable to propose novel features or explore other synergistic approaches based on multiple data sources for various forest types. 

The second limitation is that the underestimation of high AGB values and the overestimation of low AGB values were not eliminated from the wall-to-wall prediction map, although the CNN model had good efficiency and high accuracy according to model evaluation results. Data saturation might be responsible for this phenomenon and lead to a much lower average of AGB estimates of the entire study area than those values in similar studies [57,69,104,105]. The high risks of overfitting resulted from the data-driven models could be another possible reason for the big discrepancy between model evaluation results and final wall-to-wall prediction. Thus, the development of models with good generalizability in the estimation of biomass and the interpretation of the physical meaning of models are strongly recommended in further research [17]. 

In addition, the model evaluation procedure based on leave-one-out cross-validation may be another incentive for the high accuracy of the CNN model using reference data. Leave-one-out cross-validation is a special case of K-fold cross-validation where the number of folds equals the number of records in the data set [106]. Since the evaluated model is applied once for each record, using all other records as a training set and the selected record as a single-item test set, it could tend to yield higher accuracy due to overfitting compared to ten-fold cross-validation, for example, which only uses 90% records to train the model. However, the quantitative effects of different cross-validation procedures on AGB estimations still need to be further investigated. Sometimes, it could be a big difference between the accuracy of the model evaluation procedure using reference data and wall-to-wall prediction values. Thus, besides the traditional model evaluation procedure, we strongly suggest assessing the spatial distribution of AGB estimates based on a wall-to-wall prediction map and distribution of AGB estimates based on histogram compared to existed data.

The AGB estimation in this study was based on an area-based approach (ABA) that develops models to relate AGB with features derived from remotely sensed data at a plot level and apply the models over the whole study area [17]. The fixed plots of continuous forest resources inventory obtained in 2016 had an area of 20 m ×30 m with the geolocation error of 5 m, while the pixel size of Landsat 8 was 30 m × 30 m. Thus, geolocation mismatch between remotely sensed data (i.e., Landsat 8 imagery) and field measurements is another source of uncertainty of AGB estimation [107]. Fortunately, the large plot size (i.e., 195) in this study could greatly decrease the geolocation errors according to [107]: the geolocation errors will be stabilized below 5 m with 20 measurement points and below 3 m with 50 measurement points. Another drawback of this study is the lack of assessing biomass uncertainty based on ABA. It is difficult for AGB estimation using ABA to understand biomass uncertainties at different spatial scales [108]. In recent years, with the development of automatic individual tree crown delineation algorithms in precise forestry (e.g., [109,110]), the AGB estimation based on individual-tree-based approach (ITA) has received more and more attention because field data are needed only for a sample of trees instead of a sample of plots or stands [17]. In addition, ITA allows AGB estimation of tree-level, plot-level, and propagation of errors in an up-scaling framework [108]. Thus, it is appealing and worth estimating AGB based on ITA for a large-scale forest and quantifying its uncertainty from tree-level to plot-level then to stand-level in an up-scaling framework in subsequent research.

## 5. Conclusions

Accurate quantification of AGB plays a vital role in forest carbon sequestration in the context of climate change. In this study, we investigated the effects of different synergistic approaches of features and ensemble learning algorithms on AGB estimation of natural secondary forests of northeastern China based on ALS and Landsat 8 OLI imagery. It is conducive to combine active and passive data to improve the accuracy of AGB estimation. Unlike the previous study implemented in southeastern China [48], we found that *COLI*2 features are more effective in AGB estimation than *COLI*1 features for the NSFs. Sometimes, it might be more convenient and efficient to adopt the simple combination of the untransformed features (e.g., the optimal Landsat 8 features + BLV) than the novel features (i.e., *COLI*1 or *COLI*2), especially for NSFs of northeastern China. The CNN model was much superior to multiple linear regression and other classic machine learning algorithms (i.e., ELM, BP, RegT, RF, SVR, KNN) no matter of feature sets, and reached the highest accuracies (*R*^2^ = 0.99, *RMSE* = 6.85, *rRMSE* = 0.04, *MAE* = 2.95, *MAPE* = 1.02, *PM* = 0.03) when optimal ALS and Landsat 8 features and all *COLI*2 (Feature 1 + 2 + 5) was applied. Ensemble learning algorithms (SG(RF), SG(SVR), SG(KNN), SG(CNN)) that took advantage of the good and stable predictions from the base models (i.e., RF, SVR, KNN, CNN) greatly improved the accuracy of AGB and had stronger generalization ability compared to its corresponding base model. The ensemble learning algorithm is exceedingly adept to train weaker learners to strong learners, especially when applying heterogeneous ensemble strategy. The SG model with CNN meta-model performed best (*R*^2^ = 0.99, *RMSE* = 2.02, *rRMSE* = 0.01, *MAE* = 0.87, *MAPE* = 0.73, *PM* = 0.02) with the feature combination of the optimal ALS and Landsat 8 features and all *COLI*2 (Feature 1 + 2 + 5) in this study. However, considering both the efficiency (i.e., runtime) and accuracy, a wall-to-wall AGB prediction map of Maoershan was generated using the CNN model and Feature 1 + 2 + 5, instead of the SG(CNN) model. The average and standard deviation of the estimated AGB of Maoershan Experimental Forest Farm in 2015 was 59.9 Mg/ha and 48.69 Mg/ha, respectively, ranging from 0 to 491.04 Mg/ha. The lower average value than that of similar studies for northeastern China maybe because of the different study areas, data saturation, overfitting of the algorithm, and leave-one-out cross-validation. Estimating data saturation, developing advanced algorithms, understanding the effects of the different cross-validation procedures, and quantifying the sources of error are still fundamental and significant to AGB estimation at all levels.

## Figures and Tables

**Table 1:** Estimated parameters (*a* and *b*) of the allometric growth models of different species applied in this study.

Vegetation Types	Latin Names of Species	*a*	*b*
Deciduous trees	*Acer mono Maxim.*	0.318	2.081
	*Ulmus pumila L.*	0.350	1.995
	*Populus davidiana Dode*	0.078	2.512
	*Betula platyphylla*	0.313	2.114
	*Quercus mongolica Fisch. ex Ledeb.*	0.097	2.501
	*Tilia mongolica Maxim*	0.083	2.422
	*Fraxinus mandshurica Rupr./Juglans mandshurica Maxim/Phellodendron amurense Rupr.*	0.268	2.118
Coniferous trees	*Larix olgensis Henry*	0.168	2.248
	*Pinus koraiensis Sieb.et Zucc.*	0.082	2.426
	*Picea asperata Mast.*	0.067	2.517
	*Larix olgensis Henry* ^1^	0.222	2.174
	*Pinus koraiensis Sieb.et Zucc.* ^1^	0.206	2.117
	*Pinus sylvestris* var. *mongolica Litv.* ^1^	0.080	2.440
Understory	*Acer ginnala*	0.527	2.217
	*Syringa reticulata* var. *amurensis*	0.395	2.300
	*Padus asiatica*	0.090	2.696
	*Rhamnus yoshinoi*	0.169	2.555
	Arbor-like mixed species ^2^	0.182	2.487

^1^ Represents plantations; otherwise are natural forests. ^2^ represent arbor-like mixed species of understory that do not have a specific Latin name.

**Table 2:** Feature experiments designed in this study.

Experiment	Data Source	Number of Features ^1^	Details
I	ALS	9	Feature 1: Optimal ALS features
	Landsat 8	9	Feature 2: Optimal Landsat 8 features
	ALS + Landsat 8	18	Feature 1 + 2: Optimal ALS and Landsat 8 features
II	ALS + Landsat 8	9	Feature 4: All *COLI*1 ^2^
		9	Feature 5: All *COLI*2 ^2^
		10	Feature 2 + 3 ^3^: Optimal Landsat 8 features (9) + The best performing ALS feature (1)
III	ALS + Landsat 8	27	Feature 1 + 2 + 4: Optimal ALS features (9) + Optimal Landsat 8 features (9) + All *COLI*1 (9)
		27	Feature 1 + 2 + 5: Optimal ALS features (9) + Optimal Landsat 8 features (9) + All *COLI*2 (9)

^1^ Number of features was determined by the procedure described in Section 2.3.2. ^2^ *COLI*1 and *COLI*2 were calculated using Equations (3) and (4) described in Section 2.3.2. ^3^ Feature 3 is the best performing ALS feature.

**Table 3:** Feature Selection of ALS and Landsat 8 imagery.

ALS	Feature Descriptions	Landsat 8	Feature Descriptions
elev_mean	Mean value of height	MVI5	(B_5_ + B_4_ − B_2_)/(B_5_ + B_4_ + B_2_)
int_AII_5th	The cumulative intensity of 5% points in each pixel	B1	Band 1
elev_cv	Coefficient of variation of height	B76	B_7_/B_6_
density_7th	The proportion of returns in 7th height interval	B65	B_6_/B_5_
int_max	Max of intensity	B53	B_5_/B_3_
int_AII_40th	The cumulative intensity of 40% points in each pixel	Entr_B5	Entropy of band 5
int_per_60th	60% intensity percentile	B2	Band 2
int_per_80th	80% intensity percentile	ND563	(B_5_ + B_6_ − B_3_)∙(B_5_ + B_6_ + B_3_)
int_AII_50th	The cumulative intensity of 50% points in each pixel	MVI7	(B_5_ − B_7_)/(B_5_ + B_7_)

**Table 4:** Accuracy assessment of the univariate models with AGB and each ALS feature.

ALS Features	*R* ^2^	*RMSE*	*rRMSE*	*MAE*	*MAPE*	*PM*
elev_mean	0.34	60.03	0.40	43.74	0.39	0.67
int_AII_5th	0.13	68.75	0.46	51.70	0.65	0.87
elev_cv	0.08	70.64	0.48	53.99	0.66	0.92
density_7th	0.05	71.89	0.48	53.27	0.87	0.95
int_max	0.19	66.41	0.45	49.03	0.64	0.81
int_AII_40th	0.20	65.97	0.44	49.85	0.63	0.80
int_per_60th	0.17	66.89	0.45	50.68	0.64	0.83
int_per_80th	0.17	66.94	0.45	50.51	0.66	0.83

**Table 5:** Accuracy assessment of classic machine learning algorithms with three sets of features designed in experiment I.

Features	Algorithm ^1^	*R* ^2^	*RMSE*	*rRMSE*	*MAE*	*MAPE*	*PM*
Optimal ALS features (Feature 1)	MLR	0.31	52.76	0.37	41.09	38.37	0.67
	ELM	0.31	56.79	0.40	42.61	35.49	0.69
	BP	0.28	61.01	0.42	44.37	36.01	0.71
	RegT	0.21	71.95	0.47	58.55	42.66	1.11
	RF	0.29	61.84	0.41	45.80	37.08	0.72
	SVR	0.40	57.84	0.38	39.32	32.35	0.66
	KNN	0.31	60.95	0.4	45.21	35.36	0.81
	CNN	0.49	51.54	0.34	37.31	30.82	0.41
Optimal Landsat 8 features (Feature 2)	MLR	0.17	66.36	0.47	58.08	44.31	1.05
	ELM	0.12	71.64	0.48	59.73	41.40	1.21
	BP	0.13	68.58	0.49	57.19	42.76	1.04
	RegT	0.14	66.24	0.48	58.59	42.53	0.89
	RF	0.15	67.33	0.44	50.69	43.39	0.92
	SVR	0.07	70.31	0.46	51.65	47.28	1.14
	KNN	0.11	68.95	0.45	52.91	43.31	0.84
	CNN	0.27	62.54	0.41	47.16	43.08	0.72
Optimal ALS and Landsat 8 features (Feature 1 + 2)	MLR	0.25	63.48	0.40	47.21	42.34	0.94
	ELM	0.30	57.49	0.38	42.91	36.42	0.78
	BP	0.29	55.65	0.39	43.4	37.87	0.72
	RegT	0.24	60.86	0.45	55.07	39.18	0.87
	RF	0.28	61.91	0.41	45.36	39.28	0.91
	SVR	0.39	57.8	0.38	39.19	31.3	0.77
	KNN	0.22	65.37	0.43	48.6	34.69	1.07
	CNN	0.97	12.6	0.08	6.43	4.02	0.13

^1^ MLR- multiple linear regression; ELM—extreme learning machine; BP—back propagation; RegT—regression tree; RF—random forest; SVR—support vector regression; KNN—k-nearest neighbor regression; CNN—convolutional neural networks

**Table 6:** Accuracy assessment of classic machine learning algorithms with three sets of features designed in experiment II.

Features	Algorithm	*R* ^2^	*RMSE*	*rRMSE*	*MAE*	*MAPE*	*PM*
All *COLI*1 (Feature 4)	MLR	0.34	59.50	0.39	45.08	34.07	0.61
	ELM	0.31	59.25	0.41	44.27	37.7	0.66
	BP	0.30	57.34	0.38	45.68	39.39	0.68
	RegT	0.28	62.62	0.43	50.22	45.45	0.72
	RF	0.32	60.14	0.40	43.27	35.55	0.62
	SVR	0.24	69.91	0.46	51.13	43.78	0.85
	KNN	0.26	62.58	0.41	46.3	38.39	0.69
	CNN	0.5	51.06	0.34	38.27	30.48	0.54
All *COLI*2 (Feature 5)	MLR	0.22	61.49	0.48	50.12	39.34	0.72
	ELM	0.25	64.35	0.47	51.07	40.81	0.75
	BP	0.30	62.14	0.47	50.39	38.24	0.78
	RegT	0.24	67.07	0.49	52.41	43.93	0.79
	RF	0.24	63.98	0.42	46.28	39.73	0.74
	SVR	0.26	67.69	0.45	49.05	38.71	0.78
	KNN	0.25	63.51	0.42	47.3	40.05	0.71
	CNN	0.66	42.42	0.28	29.71	22.16	0.45
Optimal Landsat 8 features + The best-performing ASL feature (Feature 2 + 3)	MLR	0.33	60.14	0.40	44.45	40.76	0.70
	ELM	0.29	64.26	0.43	48.39	42.59	0.69
	BP	0.30	63.8	0.41	50.11	44.01	0.70
	RegT	0.25	64.14	0.45	52.34	45.53	0.74
	RF	0.28	62.29	0.41	45.62	41.69	0.71
	SVR	0.29	62.25	0.41	42.00	40.21	0.82
	KNN	0.24	63.38	0.42	46.95	39.24	0.69
	CNN	0.88	24.48	0.16	10.19	7.23	0.24

**Table 7:** Accuracy assessment of classic machine learning algorithms with two sets of features designed in experiment III.

Features	Algorithm	*R* ^2^	*RMSE*	*rRMSE*	*MAE*	*MAPE*	*PM*
Optimal ALS + Landsat 8 features + All *COLI*1 (Feature 1 + 2 + 4)	MLR	0.32	60.50	0.40	45.08	36.07	0.68
	ELM	0.28	63.26	0.42	44.15	37.84	0.81
	BP	0.31	58.71	0.37	40.30	36.98	0.65
	RegT	0.28	62.07	0.42	42.29	38.51	0.79
	RF	0.31	60.32	0.41	43.41	39.26	0.73
	SVR	0.39	57.74	0.39	38.05	35.31	0.66
	KNN	0.29	61.11	0.44	42.87	36.47	0.69
	CNN	0.92	12.02	0.09	11.37	8.3	0.11
Optimal ALS + Landsat 8 features + All *COLI*2 (Feature 1 + 2 + 5)	MLR	0.33	59.38	0.42	44.27	39.50	0.70
	ELM	0.29	61.67	0.43	47.09	40.34	0.81
	BP	0.32	57.74	0.42	48.29	41.60	0.72
	RegT	0.33	65.59	0.42	49.26	42.17	0.83
	RF	0.31	60.61	0.40	44.69	31.08	0.69
	SVR	0.42	56.82	0.37	38.76	29.39	0.68
	KNN	0.32	59.83	0.39	44.34	37.3	0.64
	CNN	0.99	6.85	0.04	2.95	1.02	0.03

**Table 8:** Accuracy assessment of ensemble learning algorithms with three sets of features designed in experiment I.

Features	Algorithm	*R* ^2^	*RMSE*	*rRMSE*	*MAE*	*MAPE*	*PM*
Optimal ALS features (Feature 1)	SG(RF)	0.20	65.38	0.43	50.66	42.35	1.03
	SG(SVR)	0.24	63.98	0.42	45.75	41.03	0.92
	SG(KNN)	0.19	66.07	0.44	50.70	42.22	1.24
	SG(CNN)	0.61	45.42	0.30	31.59	24.28	0.37
Optimal Landsat 8 features (Feature 2)	SG(RF)	0.44	54.24	0.36	40.20	32.47	0.57
	SG(SVR)	0.45	54.36	0.36	38.85	34.59	0.65
	SG(KNN)	0.44	54.34	0.36	40.37	32.08	0.53
	SG(CNN)	0.76	35.28	0.23	24.29	18.17	0.26
Optimal ALS and Landsat 8 features (Feature 1 + 2)	SG(RF)	0.93	18.04	0.12	8.78	6.30	0.17
	SG(SVR)	0.97	12.13	0.08	5.70	4.70	0.14
	SG(KNN)	0.9	24.27	0.16	16.76	15.09	0.15
	SG(CNN)	0.97	10.95	0.07	6.58	5.06	0.03

**Table 9:** Accuracy assessment of ensemble learning algorithms with three sets of features designed in experiment II.

Features	Algorithm	*R* ^2^	*RMSE*	*rRMSE*	*MAE*	*MAPE*	*PM*
All *COLI*1 (Feature 4)	SG(RF)	0.38	57.84	0.38	41.72	33.69	0.68
	SG(SVR)	0.48	52.83	0.35	38.69	32.08	0.62
	SG(KNN)	0.36	58.5	0.39	43.04	34.36	0.71
	SG(CNN)	0.63	43.78	0.29	31.86	25.13	0.49
All *COLI*2 (Feature 5)	SG(RF)	0.64	43.13	0.28	30.66	23.11	0.48
	SG(SVR)	0.64	43.28	0.28	31.09	28.28	0.47
	SG(KNN)	0.60	45.85	0.30	32.74	27.00	0.51
	SG(CNN)	0.50	51.31	0.34	36.80	27.66	0.50
Optimal Landsat 8 features + The best-performing ALS feature (Feature 2 + 3)	SG(RF)	0.86	26.94	0.18	14.22	10.66	0.24
	SG(SVR)	0.88	24.61	0.16	10.13	10.25	0.29
	SG(KNN)	0.79	34.06	0.23	22.46	17.88	0.31
	SG(CNN)	0.86	26.45	0.17	14.76	10.35	0.2

**Table 10:** Accuracy assessment of ensemble learning algorithms with two sets of features designed in experiment III.

Features	Algorithm	*R* ^2^	*RMSE*	*rRMSE*	*MAE*	*MAPE*	*PM*
Optimal ALS + Landsat 8 features + All *COLI*1 (Feature 1 + 2 + 4)	SG(RF)	0.95	15.35	0.11	12.44	9.34	0.14
	SG(SVR)	0.71	58.84	0.38	24.02	15.76	0.49
	SG(KNN)	0.86	57.00	0.38	21.38	15.49	0.38
	SG(CNN)	0.97	12.35	0.08	2.02	1.07	0.03
Optimal ALS + Landsat 8 features + All *COLI*2 (Feature 1 + 2 + 5)	SG(RF)	0.98	10.13	0.06	2.48	1.98	0.10
	SG(SVR)	0.95	4.10	0.18	3.20	2.34	0.08
	SG(KNN)	0.96	15.76	0.10	9.04	8.28	0.17
	SG(CNN)	0.99	2.02	0.01	0.87	0.73	0.02

**Table 11:** The runtime of all algorithms with the combination of the optimal ALS and Landsat 8 features, and all COLI2 (Feature 1 + 2 + 5).

Classic Algorithms	Runtime (s)	SG Algorithms	Runtime (s)
MLR	1.2	SG(RF)	8168
ELM	45	SG(SVR)	7798
BP	38	SG(KNN)	7794
RegT	24	SG(CNN)	15170
RF	382		
SVR	12		
KNN	8		
CNN	7384		

## Data Availability

The data presented in this study are available on request from the corresponding author.

## Appendix A

**Table A1:** The 101 features extracted from ALS data in this study.

Feature Group	Feature Name	Feature Descriptions [111]
Forest features ^1^ (3 features)	CC	Canopy cover: *CC* = *N*_veg_/*N*
	G	Gap fraction: *G* = *N*’/*N*
	LAI	Leaf area index: $LAI=-\cos\left(A\right)\cdot \ln\left(G\right)/k$
Elevation features (46 features) ^2^	elev_AAD	Average absolute deviation of elevation: $\sum_{i=1}^{n}\left(\left|Z_{i}-\bar{Z}|\right.\right)/n$
	elev_CRR	Canopy relief ratio of elevation: ($\bar{Z}$ − *Z*_min_)/(*Z*_max_ + *Z*_min_)
	elev_AIH_*i*th	The cumulative height of *i*% points in each pixel is the AIH of the pixel, *i* = 1%, 5%, 10%, 20%, 25%, 30%, 40%, 50%, 60%, 70%, 75%, 80%, 90%, 95%, 99%
	elev_AIH_IQ	AIH interquartile distance: AIH75%–AIH25%
	elev_GM_2	Generalized means for the 2nd power: $\sqrt[{2}]{\sum_{i=1}^{n}Z_{i}^{3}/n}$
	elev_GM_3	Generalized means for the 3rd power: $\sqrt[{3}]{\sum_{i=1}^{n}Z_{i}^{3}/n}$
	elev_cv	Coefficient of variation of elevation: *Z*_std_/$\bar{Z}$×100%
	elev_IQ	Elevation percentile interquartile distance: Elev75%–Elev25%
	elev_kurt	Kurtosis of elevation
	elev_MMAD	Median of median absolute deviation of elevation
	elev_max	Maximum of elevation
	elev_min	Minimum of elevation
	elev_mean	Mean of elevation
	elev_med	Median of elevation
	elev_per_*i*th	*i*th elevation percentiles, *i* = 1%, 5%, 10%, 20%, 25%, 30%, 40%, 50%, 60%, 70%, 75%, 80%, 90%, 95%, 99%
	elev_skew	Skewness of elevation
	elev_std	Standard deviation of elevation
	elev_var	Variance of elevation
Density features (10 features)	density_*i*th	The proportion of returns in *i*th height interval, *i* = 1–10
Intensity features (42 features) ^3^	int_AAD	Average absolute deviation of intensity: $\overset{n}{\underset{i=1}{\sum }}\left(\left|I_{i}-\bar{I}|\right.\right)/n$
	int_cv	Coefficient of variation of intensity: *I*_std_/$\bar{I}$×100%
	int_AII_*i*th	The cumulative intensity of X% points in each pixel is the AII of the pixel, *i* = 1%, 5%, 10%, 20%, 25%, 30%, 40%, 50%, 60%, 70%, 75%, 80%, 90%, 95%, 99%
	int_kurt	Kurtosis of intensity
	int_MMAD	Median of median absolute deviation of intensity
	int_max	Maximum of intensity
	int_min	Minimum of intensity
	int_mean	Mean of intensity
	int_med	Median of intensity
	int_per_*i*th	*i*th intensity percentiles, *i* = 1%, 5%, 10%, 20%, 25%, 30%, 40%, 50%, 60%, 70%, 75%, 80%, 90%, 95%, 99%
	int_skew	Skewness of intensity
	int_std	Standard deviation of intensity
	int_var	Variance of intensity
	Int_IQ	Intensity percentile interquartile distance: Int75%–Int25%

^1^ *N_veg_*: point number of vegetation; *N*: the total return number; *N’*: the number of ground points whose elevation is lower than the height threshold of 2m for separating ground and tree points; *A*: average scanning angle; *k*: extinction coefficient, which is closely related to the leaf inclination angle distribution of the canopy. ^2^ n is the number of points in a pixel; *Z_i_*: the elevation of *i* point within a pixel, $\bar{Z}$, *Z*_min_, *Z*_max_, *Z*_std_ are the average, minimum, maximum, and standard deviation of elevation of all points within a pixel, respectively; AIH75% and AIH25% represents the 75% and 25% AIH statistical layer, respectively. ^3^ *I**_i_*: the elevation of *i* point within a pixel, $\bar{I}$, *I*_min_, *I*_max_, *I*_std_ are the average, minimum, maximum, and standard deviation of intensity of all points within a pixel, respectively; Int75% and Int25% are 75% and 25% intensity statistical layer, respectively.

**Table A2:** The 98 spectral features extracted from Landsat 8 OLI imagery in this study.

Feature Group	Feature Name	Feature Descriptions
Original bands (7 features)	Bi ^1^	Band1–7 of Landsat 8 OLI image
Band combination (10 features)	Albedo	0.246 B_2_ + 0.146 B_3_ + 0.191∙B_4_ + 0.304∙B_5_ + 0.105∙B_6_ + 0.008∙B_7_ [112]
	B4/Albedo	B_4_/(0.246∙B_2_ + 0.146∙B_3_ + 0.191∙B_4_ + 0.304∙B_5_ + 0.105∙B_6_ + 0.008∙B_7_) [112,113]
	B24	B_2_/B_4_ [113]
	B74	B_7_/B_4_ [113]
	B76	B_7_/ B_6_ [113]
	B547	B_5_∙B_4_/B_7_ [113]
	B65	B_6_/B_5_ [113]
	B345	B_3_∙B_4_/B_5_ [113]
	B53	B_5_/B_3_ [113]
	VIS234	B_2_ + B_3_ + B_4_ [113]
GLCM features ^2^ (56 features)	Mean_B_i_	Mean of each band
	Var_B_i_	Variance of each band
	Hom_B_i_	Homogeneity of each band
	Cont_B_i_	Contrast of each band
	Diss_B_i_	Dissimilarity of each band
	Entr_B_i_	Entropy of each band
	Sec_B_i_	Second moment of each band
	Corr_B_i_	Correlation of each band
Image enhancement features (10 features)	Bright	Brightness from tasseled cap transformation: 0.3521∙B_2_ + 0.3899∙B_3_ + 0.3825∙B_4_ + 0.6985∙B_5_ + 0.2343∙B_6_ + 0.1867∙B_7_ [114]
	Green	Greenness from tasseled cap transformation: −0.3301∙B_2_−0.3455∙B_3_−0.4508∙B_4_ + 0.6970∙B_5_−0.0448∙B_6_−0.2840∙B_7_ [114]
	Wet	Wetness from tasseled cap transformation: 0.2651∙B_2_ + 0.2367∙B_3_ + 0.1296∙B_4_ + 0.059∙B_5_−0.7506∙B_6_−0.5386∙B_7_ [114]
	PC1	The first principal component from principal component analysis (PCA): 0.111∙B_3_ + 0.870∙B_5_ + 0.423∙B_6_ + 0.192∙B_7_
	PC2	The second principal component from PCA: 0.198∙B_1_ + 0.217∙B_2_ + 0.267∙B_3_ + 0.376∙B_4_−0.436∙B_5_ + 0.430∙B_6_ + 0.571∙B_7_
	PC3	The third principal component from PCA: 0.295∙B_1_ + 0.324∙B_2_ + 0.398∙B_3_ + 0.473∙B_4_ + 0.183∙B_5_−0.615∙B_6_−0.12∙B_7_
	MNF1	The first band of minimum noise fraction rotation (MNF): −0.2632∙B_1_−0.3528∙B_2_−0.0737∙B_3_−0.0618∙B_4_−0.7457∙B_5_ −0.4898∙B_6_ + 0.031∙B_7_
	MNF2	The second band of MNF: −0.0441∙B_1_−0.0781∙B_2_ − 0.1869∙B_3_ − 0.0389∙B_4_ − 0.7523∙B_5_ − 0.4280∙B_6_ − 0.4542∙B_7_
	MNF3	The third band of MNF: −0.2387∙B_1_ − 0.2230∙B_2_ + 0.0947∙B_3_ − 0.0195∙B_4_ + 0.5277∙B_5_ + 0.7731∙B_6_ − 0.0885∙B_7_
	MNF4	The fourth band of MNF: 0.0199∙B_1_ − 0.00013∙B_2_ − 0.01021∙B_3_ − 0.1027∙B_4_ − 0.4377∙B_5_ − 0.69145∙B_6_ − 0.565∙B_7_
Vegetation indices (15 features)	NDVI	Normalized vegetation index 1: (B_5_ − B_4_)/(B_5_ + B_4_) [113]
	RVI	Ratio vegetation index: B_5_/B_4_ [113]
	DVI	Difference vegetation index: B_5_ − B_4_ [113]
	EVI	Enhanced vegetation index: 2.5∙(B_5_ − B_4_)/(B_5_ + 6∙B_4_ − 7.5∙B_2_ + 1) [113]
	MSAVI	Modified soil-adjusted vegetation index: [(B_5_ − B_4_)/(B_5_ + B_4_ + L)]∙(1 + L) ^3^ [115]
	ARVI	Atmospherically resistant vegetation index: (B_5_ − 2∙B_4_ + B_2_)/(B_5_ + 2∙B_4_ − B_2_) [113]
	TVI	Triangular vegetation index: $\sqrt{\left(B_{5}-B_{4}\right)/\left(B_{5}+B_{4}\right)+0.5}$ [113]
	PVI	Perpendicular vegetation index: $\sqrt{{\left(0.355\cdot B_{5}-0.149\cdot B_{4}\right)}^{2}+{\left(0.355\cdot B_{4}-0.852\cdot B_{5}\right)}^{2}}$ [113]
	MSR	$\mathrm{Modified}\;\mathrm{simple}\;\mathrm{ratio}\;\mathrm{vegetation}\;\mathrm{index}:\;\left(B_{5}/B_{4}-1\right)/\sqrt{B_{5}/B_{4}+1}$ [113]
	SLAVI	Specific leaf area vegetation index: B_5_/(B_4_ + B_7_) [113]
	MVI5	Moisture vegetation index 1: (B_5_ + B_4_ − B_2_)/(B_5_ + B_4_ + B_2_) [116]
	MVI7	Moisture vegetation index 2: (B_5_ − B_7_)/(B_5_ + B_7_) [116]
	NLI	$\mathrm{Nonlinear}\;\mathrm{index}:\;\left(B_{5}^{2}-B_{4}\right)/\left(B_{5}^{2}+{\;B}_{4}\right)$ [113]
	RDVI	$\mathrm{Renormalized}\;\mathrm{difference}\;\mathrm{vegetation}\;\mathrm{index}:\;\;\left(B_{5}-B_{4}\right)/\sqrt{B_{5}+{\;B}_{4}}$ [113]
	ND563	Normalized difference vegetation index 2: (B_5_ + B_6_ − B_3_)/(B_5_ + B_6_ + B_3_) [113]

^1^ The index *i* represents the band index (1–7). ^2^ GLCM: gray-level co-occurrence matrix. ^3^ *L* = 2∙s∙(B_5_ − B_4_)∙(B_5_ − s∙B_4_)/(B_5_ + B_4_) where s is the slope of the soil line from a plot of red versus near infrared brightness values.
